# EUS-guided biliary drainage versus ERCP for first-line palliation of malignant distal biliary obstruction: A systematic review and meta-analysis

**DOI:** 10.1038/s41598-019-52993-x

**Published:** 2019-11-12

**Authors:** Sung Yong Han, Seon-Ok Kim, Hoonsub So, Euisoo Shin, Dong Uk Kim, Do Hyun Park

**Affiliations:** 1 Division of Gastroenterology, Department of Internal Medicine, Pusan National University School of Medicine and Biomedical Research Institute, Pusan National University Hospital, Busan, Korea; 2 Department of Clinical Epidemiology and Biostatistics, University of Ulsan College of Medicine, Asan Medical Center, Seoul, Korea; 3 Division of Gastroenterology, Department of Internal Medicine, University of Ulsan College of Medicine, Asan Medical Center, Seoul, Korea; 4 Asan Medical Library, University of Ulsan College of Medicine, Seoul, Korea

**Keywords:** Bile duct cancer, Bile ducts

## Abstract

Endoscopic retrograde cholangiopancreatography (ERCP) with transpapillary metal stenting is the standard palliation method for malignant distal biliary obstruction (MDBO); however, post-ERCP pancreatitis are not uncommon. Endoscopic ultrasonography-guided biliary drainage (EUS-BD) with transmural metal stenting has emerged as an option for primary palliation of MDBO. We compared the efficacy and safety of these procedures as first-line MDBO treatment. We searched for relevant English-language articles in PubMed, Embase, and Cochrane databases. The outcomes of interest were technical success, clinical success, adverse events, stent patency, reintervention rates, and procedure time. Subgroup analysis was performed for patients without duodenal invasion (eg, endoscopically accessible papilla; EUS-choledochoduodenostomy [CDS] vs. ERCP). Ten studies (3 randomized trials and 7 retrospective studies) with 756 patients were included. The cumulative technical and clinical success rates were high for both procedures (EUS-BD: 94.8% [294/310] and 93.8% [286/305], ERCP: 96.5% [386/400] and 95.7% [377/394]). The cumulative adverse event rates were 16.3% (54/331) for EUS-BD and 18.3% (78/425) for ERCP. In subgroup analysis for patients without duodenal invasion, EUS-CDS showed similar cumulative technical and clinical success rate with ERCP (technical success rate, EUS-CDS vs. ERCP: 94.2% [146/155] vs. 97.8% [237/242]; clinical success rate, EUS-CDS vs. ERCP: 94.2% [145/154] vs. 93.0% [225/242]). The cumulative rate of adverse events for EUS-CDS and ERCP was also comparable (15.5% [24/155] for EUS-CDS and 18.6% [45/242] for ERCP). As first-line palliation of MDBO, EUS-BD was similar to ERCP in technical and clinical success and safety; however, larger randomized trials comparing EUS-CDS and ERCP in this setting with endoscopically accessible papilla may be required.

## Introduction

Endoscopic retrograde cholangiopancreatography (ERCP) has gradually evolved to include therapeutic biliary endoscopy since the introduction of endoscopic sphincterotomy in 1974^1^. ERCP-guided stenting across the stricture and through the papilla is the standard palliative treatment for malignant distal biliary obstruction (MDBO). However, ERCP is not always successful and causes various adverse events. ERCP could fail in various situations including duodenal obstruction, surgically altered anatomy, MDBO, and periampullary tumor infiltration. Post-ERCP pancreatitis (PEP) is a major adverse event in ERCP, with an incidence of 3.5% (range, 1.6–15.7%), and can cause death in severe cases^2,3^. PEP is associated with procedure-related risk factors including difficult cannulation, guidewire passage through the pancreatic duct, and pancreatic injection^2^.

To date, endoscopic ultrasonography-guided biliary drainage (EUS-BD) has been limited to use in failed ERCP cases. A recent meta-analysis showed better clinical success, fewer adverse events, and lower reintervention rates with EUS-BD than with percutaneous transhepatic biliary drainage (PTBD) as an alternative procedure^4^. Furthermore, EUS-BD may have several advantages over ERCP. EUS-BD can be easily implemented in patients with duodenal invasion, and procedure-related pancreatitis could be prevented as endoscopists do not manipulate the major papilla during EUS-guided transmural biliary drainage. EUS-BD performed by experts may require a shorter procedure time than ERCP, with use of dedicated devices^5^. Finally, EUS-BD may show longer stent patency and lower reintervention rates, as the self-expandable metal stent does not need to be placed across the stricture, preventing tumor ingrowth or overgrowth in a metal stent^5^.

A recent study showed substantially better outcomes with EUS-BD than with ERCP as primary palliation for MDBO^5^. Recent randomized and prospective studies^6–8^ also showed good results of EUS-BD in patients without duodenal invasion; however, there remains insufficient evidence to suggest EUS-BD as a primary approach^5^. Therefore, a systematic review and meta-analysis (SRMA) on this subject is needed. We conducted this study to investigate whether EUS-BD is similar to ERCP concerning efficacy and safety in the first-line treatment of MDBO.

## Methods

### Data sources and search strategy

An experienced medical librarian (E.S.) developed the search strategy according to the PRISMA (Preferred Reporting Items for SRMA) guidelines. The literature search was conducted using PubMed, Embase, and the Cochrane Central Register of Controlled Trials for articles published between January 2001 and August 2018^9^. The search terms were combinations of synonyms for biliary obstruction and biliary drainage methods, as follows: (bile duct obstruction or biliary obstruction) and (EUS or endoscopic ultrasound or EUS-guided biliary drainage) and (ERCP or endoscopic retrograde cholangiopancreatography) and (biliary drainage or biliary stent or choledochoduodenostomy [CDS] or hepaticogastrostomy [HGS]). The search was limited to English-language literature.

### Inclusion and exclusion criteria

Randomized controlled trials (RCTs) or observational studies comparing EUS and ERCP for MDBO were selected, focusing on either efficacy or safety, or both. The exclusion criteria were (1) case reports, conference reports, editorials, letters, and reviews; (2) animal studies; (3) abstracts and meta-analyses; (4) studies with no comparative data; (5) patients with special characteristics, such as surgically altered anatomy; and (6) other disease entities. Two authors (S.Y.H. and H.S.) independently assessed the eligibility of the searched articles.

### Outcome assessment

The primary outcomes were technical success and adverse events for EUS-BD and ERCP. Given the novelty of EUS-BD, we believe that detailed definitions of technical success and adverse events for primary outcomes are warranted^5,6^. Technical success was defined as placement of the metal stent as planned via ERCP or EUS^10^. The secondary outcomes were clinical success, stent patency, reintervention rates, and procedure time for EUS-BD and ERCP. Clinical success was defined as resolution of biliary obstruction clinically and as reflected by laboratory parameters, or a 75% decrease in bilirubin level at 4 weeks^4^.

### Data extraction and quality assessment

The following data were extracted: (1) study characteristics (authors, year of study, affiliation, country of origin, study design); (2) patient characteristics (sample size); (3) technical and clinical success rates; (4) adverse events, including procedure-related pancreatitis and bile peritonitis; and (5) stent patency, reintervention rates, and total procedure time. The Cochrane tool for assessing risk of bias was used for RCTs, and the Newcastle-Ottawa scale was used for assessment of observational studies. Two authors (S.Y.H. and H.S.) independently evaluated the quality of the studies, and any disagreement was resolved with consensus of a third or fourth reviewer (D.U.K. and D.H.P.).

### Data synthesis and analysis

We performed a random-effects meta-analysis to synthesize the data by pooling the results of RCTs and cohort studies. We used a random-effect model to account for variations across studies, as the subjects or interventions in these studies would have shown some differences. Treatment effect estimates were reported with pooled risk ratios (RRs) and 95% confidence intervals (CIs) for dichotomous outcomes, including technical and clinical success rates, adverse events, reintervention rates, pooled hazard ratios (HRs) and 95% CIs for stent patency, and pooled estimates of weighted mean differences and 95% CIs for total procedure time. We attempted to contact individual investigators and asked them to reanalyze existing data based on statistical parameters in pooled data. We used forest plots for primary and secondary outcomes.

Many of the included patients with duodenal stenosis were “second-line” patients who cannot undergo ERCP. This also causes unnecessary bias away from ERCP, as many patients had duodenal stenosis in the retrospective cohort (for which EUS-BD is most likely to be effective)^11^.

As usual, EUS-HGS was considered in patients with MDBO and duodenal bulb invasion (e.g., gastric outlet obstruction), periampullary duodenal invasion with compromised duodenal bulb, or surgically altered anatomy. In patients with MDBO and periampullary tumor infiltration with distal duodenal invasion, EUS-CDS was considered first^5^.

To determine the role of EUS-BD as the routine first-line option for MDBO in patients without duodenal invasion (eg, endoscopically accessible papilla), therefore, a subgroup analysis comparing EUS-CDS and ERCP in this cohort was conducted.

The I^2^ statistic was used to assess the magnitude of heterogeneity between studies. An I^2^ of 0% to <25% was considered to indicate no heterogeneity; 25% to <50%, low heterogeneity; 50% to <75%, moderate (substantial) heterogeneity; and 75% to 100%, high (considerable) heterogeneity. The potential risk of publication bias for primary outcomes was evaluated by constructing funnel plots, and asymmetry was assessed using Egger’s test. We used STATA version 11 (StataCorp, College Station, TX, USA) for all analyses.

## Results

### Search results and quality assessment

**Figure 1:**
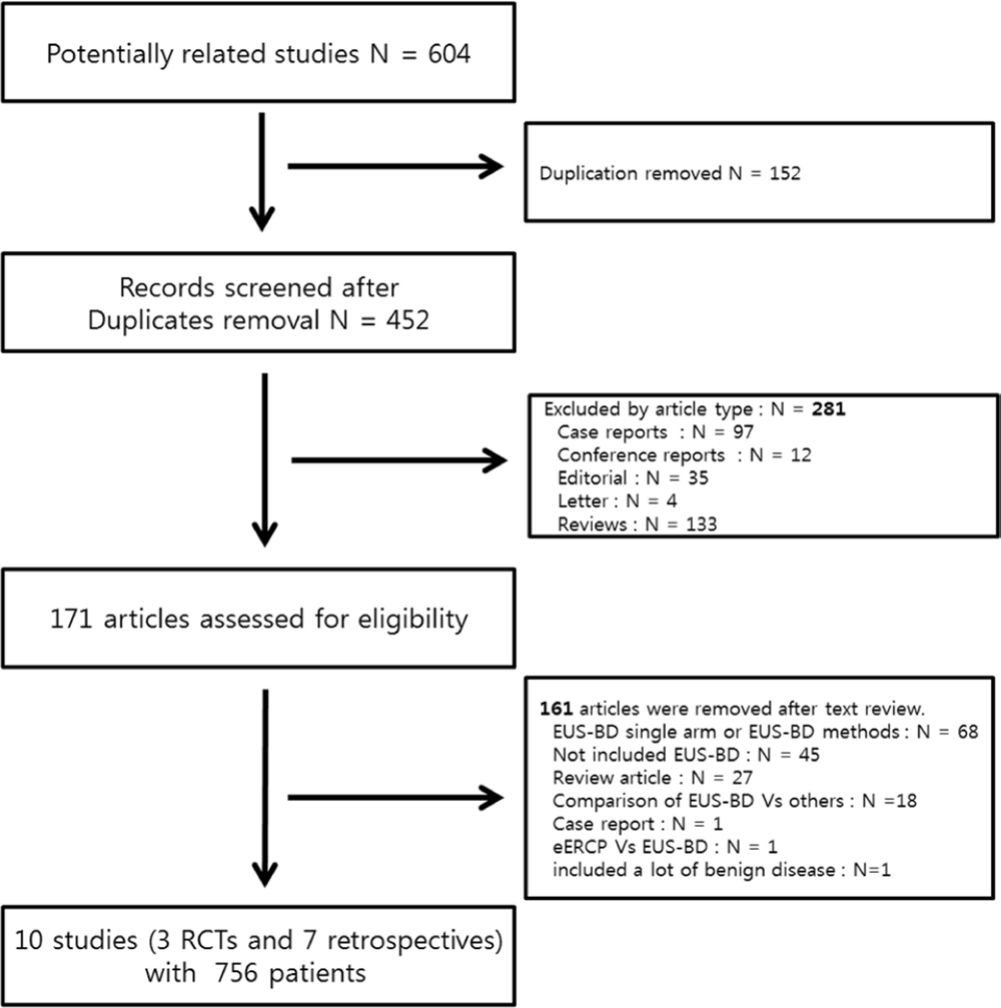
Flowchart of the study selection process.

A total 604 articles were identified. The detailed search strategy is described in Supplementary Table 1. After excluding duplicates and irrelevant articles by reviewing abstracts and titles, 171 articles remained. A full-text review was performed, and 161 articles were removed according to the exclusion criteria. The following articles were excluded: (1) studies with no comparative data (n = 68), (2) irrelevant articles that did not include EUS-BD (n = 45), (3) review articles on EUS-BD (n = 27), (4) comparison studies between EUS-BD and another method such as PTBD or surgery (n = 18), and (5) reports with < 5 cases (n = 1). Moreover, 1 article^12^ was removed as it described enteroscopy-assisted ERCP in patients with surgically altered anatomy and another study was removed as the procedure was performed mostly for benign diseases^13^. Finally, 10 articles (3 RCTs^5–7^ and 7 retrospective articles^11,14–19^) were included in the meta-analysis (Fig. 1).

Our meta-analysis of 10 studies included 756 patients, of whom 331 underwent EUS-BD and 425 underwent ERCP. Among 220 patients in 3 RCTs, 111 underwent EUS-BD and 109 underwent ERCP. For further analysis, we compared the results of EUS-CDS and ERCP in patients without duodenal invasion. Six studies^5–7,11,13,16^ reported technical/clinical success rates and adverse event rates (including bile peritonitis and pancreatitis) in patients without duodenal invasion. Some articles^11,13,16^ did not show detailed results. Therefore, we asked the authors of these studies via e-mail for the above-mentioned results. However, some retrospective studies including several cases without duodenal invasion were excluded from our analysis. Among 397 patients without duodenal invasion, 155 underwent EUS-BD and 242 underwent ERCP.

The risk of performance bias was high because none of the RCTs applied blinding of experimental and control procedures. The risk of detection bias was unclear in 1 study; however, the risk of detection bias was low in 2 studies. The risk of reporting, attrition, and selection bias was low in all RCTs. Retrospective articles were assessed with the Newcastle-Ottawa scale; 4 studies showed high quality and 3 showed moderate quality (Supplementary Table 2).

### Primary outcomes

#### Comparison of technical success rates

**Figure 2:**
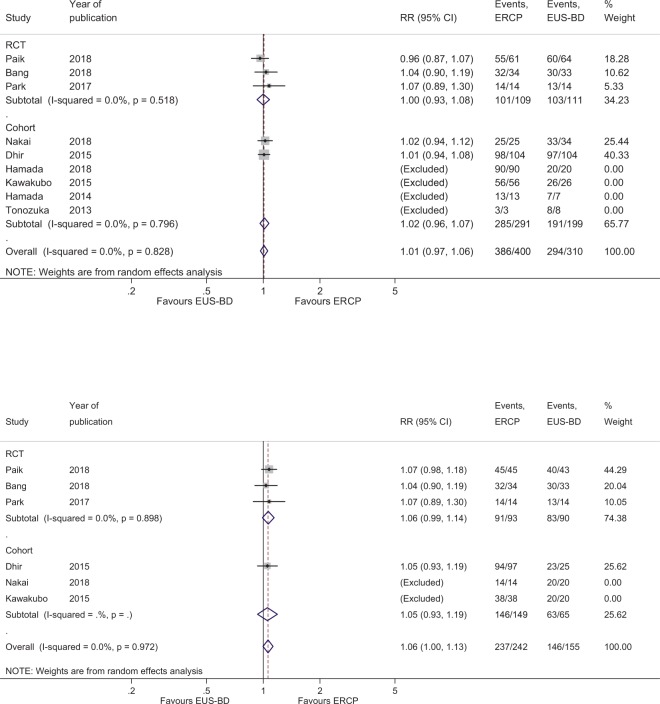
(**a**) Forest plot comparing technical success. (**b**) Forest plot comparing technical success in patients without duodenal invasion.

The overall RR for technical success between EUS-BD and ERCP was 1.01 (95% CI, 0.97–1.06; Q-test p = 0.828, I^2^ = 0%). There were no significant differences in technical success rates between RCTs and retrospective studies (Fig. 2a). The cumulative technical success rates were high in both procedures (EUS-BD: 94.8% [294/310], ERCP: 96.5% [386/400]). In subgroup analysis for patients without duodenal invasion, EUS-CDS showed similar technical success rates with ERCP (RR, 1.06; 95% CI, 1.00–1.13; Q-test p = 0.973, I^2^ = 0%) (Fig. 2b). The cumulative technical success rate in patients without duodenal invasion was 94.2% (146/155) for EUS-CDS and 97.8% (237/242) for ERCP.

#### Comparison of adverse events

**Figure 3:**
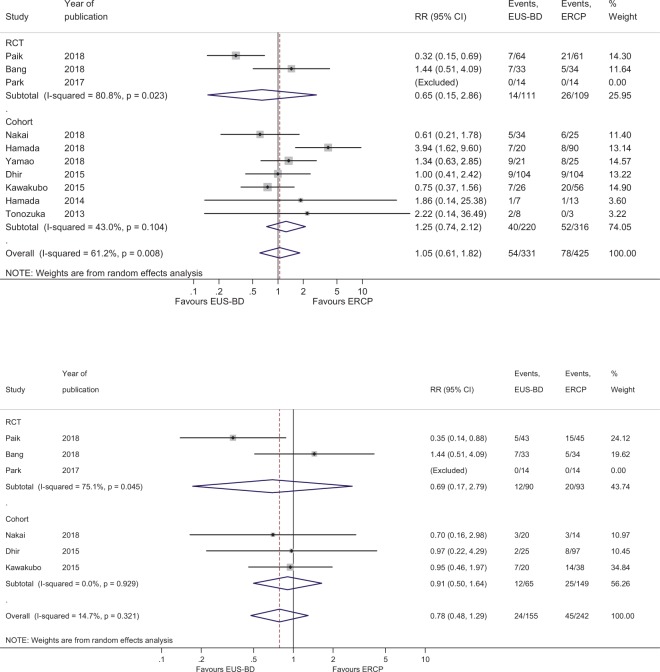
(**a**) Forest plot comparing adverse events. (**b**) Forest plot comparing adverse events in patients without duodenal invasion.

The overall RR for adverse events in EUS-BD and ERCP was 1.05 (95% CI, 0.61–1.82; Q-test p = 0.008, I^2^ = 61.2%) in all studies (Fig. 3a). The cumulative adverse event rate was 16.3% (54/331) for EUS-BD and 18.3% (78/425) for ERCP. However, in RCTs, the adverse event rates for ERCP were relatively high with considerable heterogeneity in each article. In all articles, the adverse events after EUS-BD and ERCP were as follows (EUS-BD [n]:ERCP[n]): cholangitis (17:20), pancreatitis (1:31), cholecystitis (8:13), bile peritonitis (8:0), pneumoperitoneum (3:0), abdominal pain (7:8), bleeding (4:1), stent migration (3:1), perforation (3:1), liver abscess (2:1), fever (0:3), and stent occlusion (0:3). When the incidence rates of procedure-related pancreatitis and bile peritonitis were compared for the 2 methods, there were significantly different results (RR, 0.26; 95% CI, 0.10–0.72, Q-test p = 0.619, I^2^ = 0% and RR, 5.16; 95% CI, 1.44–18.51; Q-test p = 0.984, I^2^ = 0%, respectively). The cumulative rate of procedure-related pancreatitis was 0.3% (1/331) for EUS-BD and 7.3% (31/425) for ERCP (Supplementary Fig. 1). The cumulative rate of bile peritonitis was 2.4% (8/331) for EUS-BD and 0% (0/425) for ERCP (Supplementary Fig. 2). There were no severe adverse events in the ERCP group (0%), and there was 1 severe adverse event in the EUS-BD group (1/331, 0.3%). The patient experienced peritoneal stent migration after EUS-guided HGS, and underwent surgical hepaticojejunostomy^17^.

In subgroup analysis for patients without duodenal invasion, EUS-CDS showed similar adverse event rates with ERCP (RR, 0.78; 95% CI, 0.48–1.29; Q-test p = 0.321, I^2^ = 14.7%) (Fig. 3b). The cumulative rate of adverse events was 15.5% (24/155) for EUS-CDS and 18.6% (45/242) for ERCP. The cumulative rate of procedure-related pancreatitis was 0% (0/155) for EUS-CDS and 8.7% (21/242) for ERCP. The cumulative rate of bile peritonitis was 2.6% (4/155) for EUS-CDS and 0% (0/242) for ERCP. Evaluation with Egger’s regression test did not detect any obvious asymmetric distribution or small-study effect (p = 0.646); however, the funnel plot showed slight publication bias, owing to the small number of studies included and the moderate heterogeneity (Supplementary Fig. 3). The trim and fill method was used to adjust for publication bias, and the corrected RR in 12 studies was 0.801 (95% CI, 0.446–1.441) (Supplementary Fig. 4)^20^.

### Secondary outcomes

#### Short-term results

**Figure 4:**
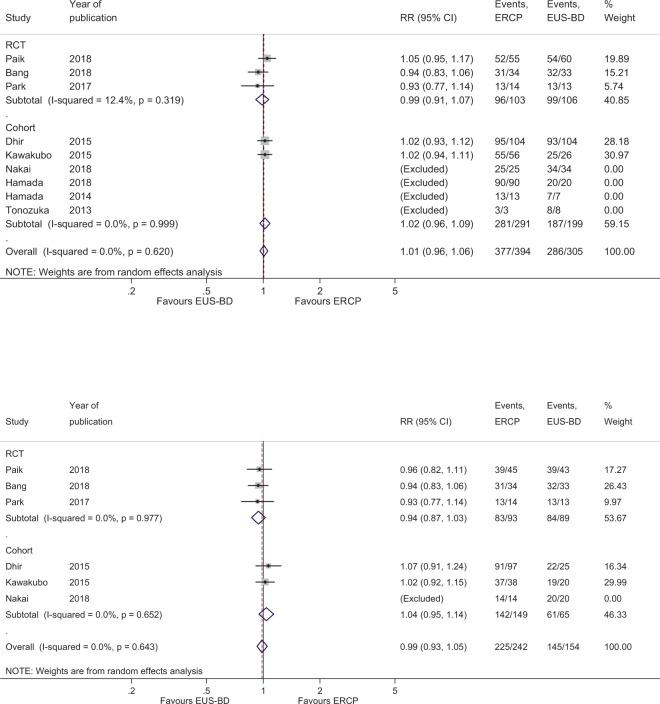
(**a**) Forest plot comparing clinical success. (**b**) Forest plot comparing clinical success in patients without duodenal invasion.

Comparison of clinical success rate and procedure time: The overall RR for clinical success between EUS-BD and ERCP was 1.01 (95% CI, 0.96–1.06; Q-test p = 0.620, I^2^ = 0%) (Fig. 4a). The cumulative clinical success rates were high in both procedures (EUS-BD: 93.8% [286/305], ERCP: 95.7% [377/394]). In subgroup analysis for patients without duodenal invasion, EUS-CDS showed similar clinical success rates with ERCP (RR, 0.99; 95% CI, 0.93–1.05; Q-test p = 0.644, I^2^ = 0.0%) (Fig. 4b). The cumulative clinical success rate in patients without duodenal invasion was 94.2% (145/154) for EUS-CDS and 93.0% (225/242) for ERCP. When compared for procedure time, the overall standard mean difference was −5.35 (95% CI, −11.81 to 1.10; Q-test p = 0.000, I^2^ = 81.2%) in favor of EUS-BD (Supplementary Fig. 5). The mean procedure time of EUS-BD was 8.31–43 min and that of ERCP was 13.1–52.6 min. Although there was no statistical significance, EUS-BD had a shorter procedure time (by about 5 min).

#### Mid-/long-term results

Reintervention rate and stent patency. The reintervention rate was compared between EUS-BD and ERCP with an overall RR of 0.82 (95% CI, 0.48–1.41; Q-test p = 0.059, I^2^ = 50.6%) (Supplementary Fig. 6). The incidence of reintervention for EUS-BD was 19.4% (38/196) and that for ERCP was 25.9% (78/291). Comparison of stent patency between procedures revealed an overall HR of 0.74 (95% CI, 0.35–1.56; Q-test p = 0.006, I^2^ = 69.4%) (Supplementary Fig. 7). EUS-BD had a tendency toward lower reintervention rates and longer stent patency than ERCP; however, there was no statistically significant difference between them.

## Discussion

This meta-analysis showed comparable results for EUS-BD and ERCP in relieving MDBO. Furthermore, EUS-CDS showed similar clinical and technical success and overall adverse event profiles with lower rates of procedure-related pancreatitis compared with ERCP in the subgroup without duodenal invasion.

EUS-BD was initially reported in 2001 by Giovannini *et al*., who performed CDS with a plastic stent^21^. It has begun to replace PTBD as a rescue method in patients with ERCP failure. A recent meta-analysis^4^ reported that EUS-BD has better results in terms of success rate, adverse event rates, and reintervention rate than PTBD. One meta-analysis^22^ of EUS-BD methods (HGS vs. CDS) compared success rates and adverse events in studies published before and after 2013. Increased success rates and decreased adverse events were observed in studies published after 2013. These findings may be due to improvements of EUS-BD accessories, better procedure organization, and greater endoscopist experience.

Recently, several retrospective and randomized studies comparing ERCP and EUS-BD as first-line options for relieving MDBO have been published^5–7,11,14–18^. The technical and clinical success rates and adverse events were comparable between the procedures. However, the sample size of each study may be underpowered^23^.

A summary of meta-analysis results with separate pooling of RCTs and retrospective studies is shown in Table 1. This meta-analysis revealed similar technical and clinical success rates for EUS-BD and ERCP (technical: 94.8% vs. 96.5%, clinical: 93.8% vs. 95.7%). The results were similar and highly homogeneous in RCTs and retrospective studies.

**Table 1:** Summary of meta-analysis results in randomized controlled trials and retrospective cohort studies.

Type	Outcomes		No. of studies	EUS-BD			ERCP			RR (95% CI)	I^2^
				n	Events	%	n	Events	%		
RCT	Technical success		3	111	103	92.79	109	101	92.66	1.00 (0.93–1.08)	0.0%
	Clinical success		3	106	99	93.40	103	96	93.20	0.99 (0.91–1.07)	12.4%
	Adverse event	Overall	3	111	14	12.61	109	26	23.85	0.65 (0.15–2.86)	80.8%
		Pancreatitis	3	111	0	0.00	109	10	9.17	0.12 (0.01–0.97)	0.0%
		Peritonitis	3	111	2	1.80	109	0	0.00	2.97 (0.32–28.04)	0.0%
	Reintervention		3	110	13	11.82	108	31	28.70	0.40 (0.23–0.71)	0.0%
Cohort	Technical success		6	199	191	95.98	291	285	97.94	1.02 (0.96–1.07)	0.0%
	Clinical success		6	199	187	93.97	291	281	96.56	1.02 (0.96–1.09)	0.0%
	Adverse event	Overall	7	220	40	18.18	316	52	16.46	1.25 (0.74–2.12)	43.0%
		Pancreatitis	7	220	1	0.45	316	21	6.65	0.33 (0.11–1.05)	0.0%
		Peritonitis	7	220	6	2.73	316	0	0.00	6.72 (1.42–31.74)	0.0%
	Reintervention		4	86	25	29.07	183	47	25.68	1.19 (0.79–1.79)	0.0%

RR > 1: Favors ERCP.

RR < 1: Favors EUS-BD.

EUS-BD, endoscopic ultrasonography-guided biliary drainage; ERCP, endoscopic retrograde cholangiopancreatography; RR, risk ratio; CI, confidence interval; RCT, randomized controlled trial.

In cases of biliary obstruction without duodenal invasion (eg, endoscopically accessible papilla), EUS-CDS showed similar technical and clinical success rate with ERCP (technical success rate, EUS-CDS vs. ERCP: 94.2% vs. 97.8%; clinical success rate, EUS-CDS vs. ERCP: 94.2% vs. 93.0%). Duodenal invasion was found in 13–20% of patients with pancreatic or biliary tract cancer^24^, and duodenal invasion is an important cause of failure to access the ampulla with duodenoscopy, especially in duodenal obstruction proximal to or involving the ampulla. EUS-BD does not need to pass the duodenum, and many cases of biliary obstruction with duodenal invasion were located below the superior duodenal angle or the second portion of the duodenum. In this circumstance, EUS-BD was predicted to have better success rates than ERCP in duodenal invasion.

For EUS-BD to serve as a primary method, there should be meaningful results in comparison with ERCP in the absence of duodenal invasion. Our results showed comparable outcomes between EUS-CDS and ERCP in the subgroup without duodenal invasion. Furthermore, EUS-CDS is more advantageous with respect to adverse event profiles such as PEP even if there is no bile leak compared with ERCP stenting. EUS-BD has potential benefit especially in preoperative biliary drainage because it can prevent PEP, which is a major cause of delay during the preoperative period. Further, some studies proved that preoperative EUS-CDS causes no significant problem in the surgical procedure^7,25^. Given that relatively few patients with accessible papilla underwent EUS-CDS and ERCP in previous studies, further larger RCT comparing EUS-CDS with a dedicated device and ERCP-guided transpapillary stenting for resectable pancreatic cancer with accessible papilla may be warranted, to determine whether EUS-CDS for preoperative biliary drainage has a potential benefit in preventing PEP and to determine its clinical impact on surgical outcomes compared with ERCP-guided transpapillary stenting.

A recent multicenter study^5^ revealed that EUS-BD has longer stent patency, fewer reinterventions, and shorter procedure times than ERCP for the primary palliation of MDBO. The authors attributed these results to the use of dedicated devices and substantial endoscopist experience. Dedicated stent introducers such as DEUS (Standard Sci Tech, Seoul, South Korea) and Hot-Axios (Boston Scientific, Natick, MA, USA) provided improved clinical results by simplifying the complex steps of EUS-BD^26^. Recently, there have been reports on the usefulness of lumen-apposing metal stent in EUS-CDS^8,25^. The use of these new devices could result in better success rates and lower adverse event rates. Oh *et al*.^27^ reported that substantial endoscopist experience in EUS-HGS contributed to shorter procedure time, as well as increased success and decreased adverse event rates. Given the technical feasibility of EUS-CDS compared with EUS-HGS, EUS-CDS with a dedicated device may gain popularity for primary palliation of MDBO, as a viable alternative to ERCP, in the near future. Furthermore, EUS-BD resulted in relatively longer stent patency despite tumor progression because a self-expandable metallic stent is not placed across the stricture site^28^.

A direct comparison of adverse events for EUS-BD and ERCP may be difficult because there are many such circumstances and the spectrum of adverse events differs among studies. Our study showed similar overall cumulative adverse event rates for EUS-BD and ERCP (16.3% vs. 18.3%). Bile peritonitis was a major concern in EUS-BD, whereas procedure-related pancreatitis was a major concern in ERCP. In EUS-BD, the incidence of bile peritonitis was 2.4%, and 1 patient had a severe adverse event due to stent migration. In ERCP, the overall incidence of procedure-related pancreatitis was 7.3%, with no severe adverse events. However, bile peritonitis associated with EUS-BD may be reduced by the use of dedicated devices for EUS-BD^5^. PEP may be related to morbidity, mortality, and prolonged hospitalization. Although there have been various efforts to prevent PEP, it still occurs^29,30^. Thus, from the viewpoint of the higher incidence and difficult management of PEP compared with bile peritonitis, EUS-BD with dedicated devices for the prevention of bile peritonitis may be preferred as the first-line procedure for patients with a high risk for PEP.

Our study has some limitations. First, some results had high heterogeneity owing to the different analysis methods in some articles. Second, the definitions of outcomes were unclear in some studies although we contacted the corresponding authors to ask for details. Third, only 3 RCTs with relatively small numbers of cases were included. Most patients were enrolled in retrospective studies, which had a weak, heterogeneous methodology compared with RCTs. Especially, 1 study enrolled patients with failed ERCP in the EUS-BD group^11^. These data may have diluted the results of RCTs. Fourth, in this analysis, various methods of EUS-BD, including HGS and CDS, were used. Fifth, with recent developments in chemotherapy and best supportive care, patients now have better survival chances than before. However, in this analysis, most data were short-term results and long-term data were lacking. Further studies are needed to determine whether EUS-BD is appropriate for long-term survivors.

In summary, EUS-BD has technical and clinical success rates similar to those of ERCP. The adverse event rates were also similar despite different adverse event entities in the primary palliation of MDBO. EUS-BD showed a significantly lower incidence of PEP but a higher incidence of bile peritonitis than ERCP. In patients without duodenal invasion, EUS-CDS was similar to ERCP in terms of technical/clinical success rates and adverse event profiles in the first-line palliation of MDBO. Given the few randomized trials and the heterogeneous methodology of retrospective studies in this SRMA, further larger randomized trials comparing EUS-CDS with a dedicated device and ERCP for the primary treatment of MDBO in patients with endoscopically accessible papillae at the setting of preoperative biliary drainage or palliation may be required.

## Supplementary information

supplementary table and figure
