# Supplementary material for: EUS-guided biliary drainage versus ERCP for first-line palliation of malignant distal biliary obstruction: A systematic review and meta-analysis
Source: Sci Rep. 2019 Nov 12;9:16551. doi: 10.1038/s41598-019-52993-x (PMC6851119; doi:10.1038/s41598-019-52993-x)
Supplement: Supplementary file 1 — supplementary table and figure [file 41598_2019_52993_MOESM1_ESM.pdf]

EUS-guided biliary drainage versus ERCP for first-line palliation of malignant distal biliary obstruction: A systematic review and meta-analysis

Sung Yong Han[1*], Seon-Ok Kim[2*], Hoonsub So[3], Euisoo Shin[4], Dong Uk Kim[1], Do Hyun Park[3]

[1]Division of Gastroenterology, Department of Internal Medicine, Pusan National University School of Medicine and Biomedical Research Institute, Pusan National University Hospital, Busan, Korea

[2]Department of Clinical Epidemiology and Biostatistics, University of Ulsan College of Medicine, Asan Medical Center, Seoul, Korea

[3]Division of Gastroenterology, Department of Internal Medicine, University of Ulsan College of Medicine, Asan Medical Center, Seoul, Korea

[4]Asan Medical Library, University of Ulsan College of Medicine, Seoul, Korea

*Sung Yong Han and Seon-Ok Kim contributed equally to this work.

Running head: EUS-BD vs. ERCP for biliary obstruction

Corresponding author

Do Hyun Park, M.D., Ph.D.

Division of Gastroenterology, Department of Internal Medicine, Univeristy of Ulsan College of Medicine, Asan Medical Center, 88, Olympic-Ro 43-Gil, Songpa-gu, Seoul 138-736, Korea

Tel.: +82-2-3010-3194

Fax: +82-2-476-0824

E-mail: dhpark@amc.seoul.kr

Supplementary Material

Supplementary Table 1. Documentation of search strategies

Date: August 31, 2018

Topic/research question:

Name of researcher(s):

Librarian: Euisoo Shin

Databases:

1. PubMed
2. Embase (embase.com)
3. Cochrane Library (Wiley)

Total number of hits:

- Before deduplication: 604
- After deduplication: 452

1. PubMed

Date of Search: 08-31-2018

Search	Query	Results
#1	("bile duct"[TIAB] OR biliary[TIAB]) AND obstruct*[TIAB]	13,557
#2	"Cholestasis"[Mesh] OR Cholestas*[TIAB]	37,529
#3	#1 OR #2	45,035
#4	"Endoscopic ultrasound"[TIAB] OR "EUS"[TIAB]	11,088
#5	Endosonography[Mesh]	11,483
#6	#4 OR #5	17,356
#7	"cholangiopancreatography, endoscopic retrograde"[MeSH]	15,294
#8	(Endoscopic[TIAB] AND retrograde[TIAB] AND (cholangiopancreatograp*[TIAB] OR "cholangio-pancreatography"[TIAB])) OR "ERCP"[TIAB]	12,629
#9	#7 OR #8	20,379
#10	drainage[Mesh] OR "biliary drainage"[TIAB] OR "biliary stent"[TIAB]	58,116
#11	"choledochostomy"[MeSH] OR choledochostom*[TIAB] OR hepaticogastrostom*[TIAB]	1459
#12	#10 OR #11	59,231
#13	#3 AND #6 AND #9 AND #12	215
#14	#13 AND (("2001/01/01"[PDAT]: "3000/12/31"[PDAT]) AND English[lang])	208

2. Embase (embase.com)

Date of Search: 08-31-2018

Search	Query	Results
#1	'cholestasis'/exp OR cholestas*:ab,ti,kw	46,470
#2	((biliary OR 'bile ducts' OR 'bile duct') NEAR/6 obstruct*):ab,ti,kw	13,142
#3	#1 OR #2	52,130
#4	'endoscopic ultrasonography'/exp	26,781
#5	'endoscopic ultrasound':ab,ti,kw OR 'eus':ab,ti,kw	22,279
#6	#4 OR #5	35,702
#7	'endoscopic retrograde cholangiopancreatography'/exp	35,380
#8	((endoscopic NEAR/6 retrograde NEAR/6 (cholangiopancreatograp* OR 'cholangio-pancreatography')):ab,ti,kw) OR 'ercp':ab,ti,kw	22,298
#9	#7 OR #8	38,269
#10	'biliary tract drainage'/exp	19,868
#11	(biliary NEAR/3 (drainag* OR stent*)):ab,ti,kw	11,036
#12	choledochostom*:ab,ti,kw OR hepaticogastrostom*:ab,ti,kw	508
#13	#10 OR #11 OR #12	25,584
#14	#3 AND #6 AND #9 AND #13	716
#15	#14 AND [english]/lim AND [2001-2018]/py	690
#16	#15 NOT ('conference abstract'/it OR 'conference paper'/it OR 'conference review'/it)	368

3. Cochrane Library (Wiley)

Date of Search: 08-31-2018

Search	Query	Results
#1	MeSH descriptor: [Cholestasis] explode all trees	700
#2	Cholestas*:ab,ti,kw or (("bile duct*":ab,ti,kw or biliary:ab,ti,kw) near/6 obstruct*:ab,ti,kw)	1294
#3	#1 or #2	1527
#4	MeSH descriptor: [Endosonography] explode all trees	312
#5	Endoscopic ultrasound:ab,ti,kw or "EUS":ab,ti,kw	874
#6	#4 or #5	1057
#7	MeSH descriptor: [Cholangiopancreatography, Endoscopic Retrograde] explode all trees	598
#8	(Endoscopic:ab,ti,kw near/6 retrograde:ab,ti,kw near/6 (cholangiopancreatograp*:ab,ti,kw or "cholangio-pancreatography":ab,ti,kw)) or "ERCP":ab,ti,kw	1572
#9	#7 or #8	1572
#10	MeSH descriptor: [Drainage] explode all trees	2537
#11	biliary:ab,ti,kw near/3 (Drainag*:ab,ti,kw or stent*:ab,ti,kw)	543
#12	MeSH descriptor: [Choledochostomy] explode all trees	20
#13	choledochostom*:ab,ti,kw or hepaticogastrostom*:ab,ti,kw	41
#14	#10 or #11 or #12 or #13	3010
#15	#3 and #6 and #9 and #14	27
#16	#15 Publication Year from 2001 to 2018, in Trials	27

Supplementary Table 2. Characteristics of studies and patients

Study	Study design	Groups	No. of patients	Technical success rate (%)	Clinical success rates (%)	Adverse events (%)	Bile pancreatitis/ procedure-related peritonitis (%)	Severe adverse events (%)	Study quality
Paik[5], 2018, 4 referral centers in Korea	RCT	EUS-BD	64	94	90.0 (54/60)	10.9	0/1.6	0	High risk of performance bias
		ERCP	61	90	94.8 (52/55)	39.3	14.8/0	0	
Bang[7], 2018, USA	RCT	EUS-BD	33	90.9	97 (32/33)**	21.2	0/3	0	High risk of performance bias
		ERCP	34	94.1	91.2 (31/34)	14.7	2.9/0	0	
Park[6], 2017, Korea	RCT	EUS-BD	14	92.8	100 (13/13)	0	0/0	0	High risk of performance bias, Unclear risk of detection bias
		ERCP	14	100	92.8 (13/14)	0	0/0	0	
Nakai[19], 2018, 10 referral centers in Japan	Retrospective	EUS-BD	34	97	100	14.7	0/0	0	7 (high quality)
		ERCP	25	100	100	24	1/0	0	
Hamada[17], 2018, 16 referral centers in 4 Asian countries	Retrospective	EUS-BD	20	100	100	35	5/5	5	4 (moderate quality)
		ERCP	90	100	100	8.9	3.3/0	0	
Yamao[18], 2018, 5 referral centers in Japan	Retrospective	EUS-BD	21	95.2	90.5 (19/21)	42.9	0/4.8	0	7 (high quality)
		ERCP	25	56	52 (13/25)	32	8/0	0	
Dhir[11], 2015, 7 referral centers in 5 countries	Retrospective	EUS-BD	104	93.3	89.4 (93/104)	8.65	0/2.9	0	6 (moderate quality)
		ERCP	104	94.2	91.3 (95/104)	8.65	4.8/0	0	
Kawakubo[16], 2016, 7 referral centers in 5 countries	Retrospective	EUS-BD	26	100	96.2 (25/26)	26.9	0/3.8	0	7 (high quality)
		ERCP	56	100	98.2 (55/56)	35.7	16.1/0	0	
Hamada[15], 2014, 3 referral centers in Japan	Retrospective	EUS-BD	7	100	100	14.2	0/0	0	8 (high quality)
		ERCP	13	100	100	7.7	7.7/0	0	
Tonozuka[14], 2013, Japan	Retrospective	EUS-BD	8	100	100	25	0/0	0	6 (moderate quality)
		ERCP	3	100	100	0	0/0	0	

**In 3 patients, the procedure was changed after failure of EUS-BD.

RCT, randomized controlled trial; EUS-BD, endoscopic ultrasonography-guided biliary drainage; ERCP, endoscopic retrograde cholangiopancreatography

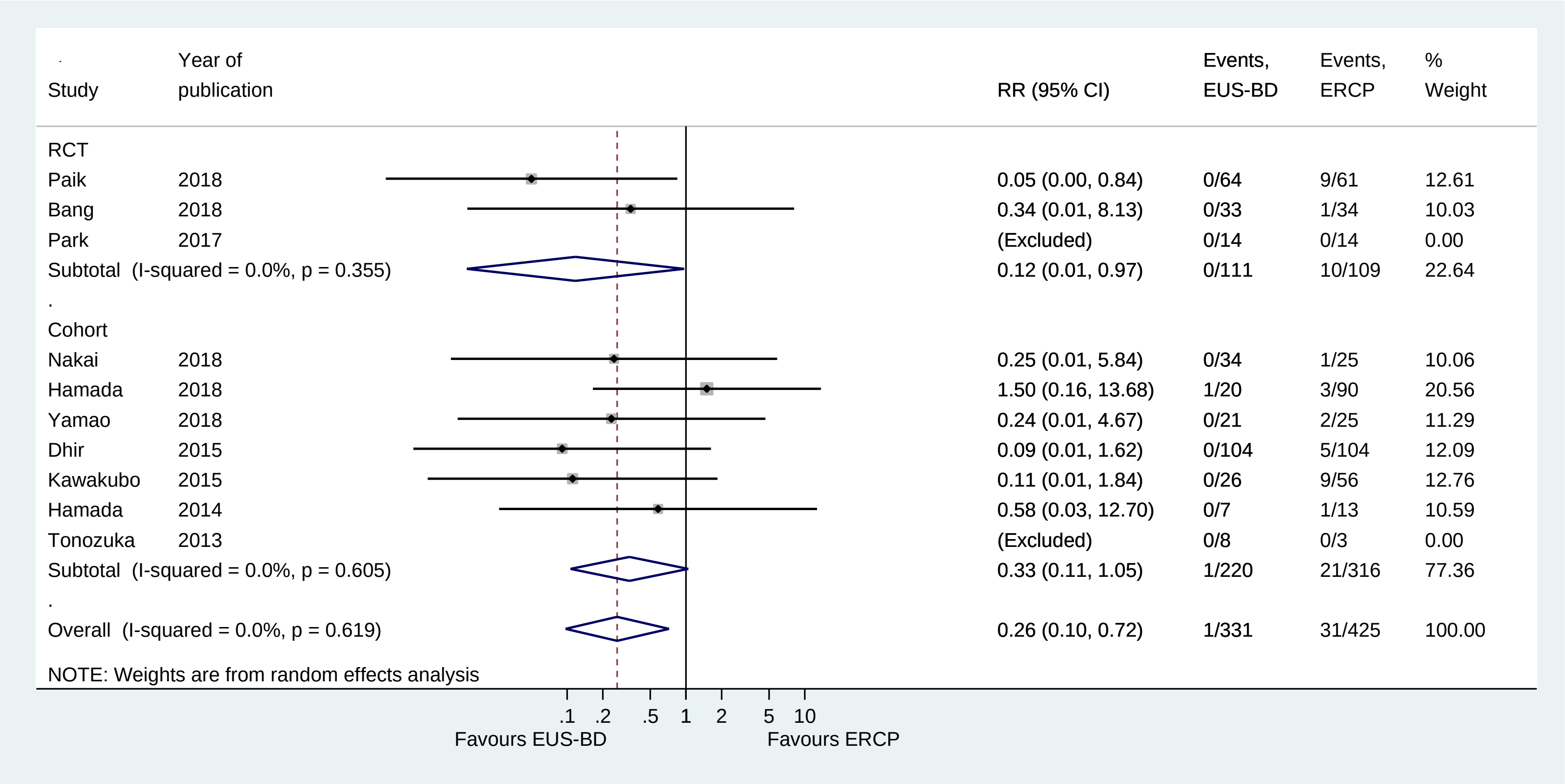

Supplementary Figure 1. Forest plot comparing procedure-related pancreatitis

RR, risk ratio; CI, confidence interval; EUS-BD, endoscopic ultrasonography-guided biliary drainage; ERCP, endoscopic retrograde cholangiopancreatography; RCT, randomized controlled trial

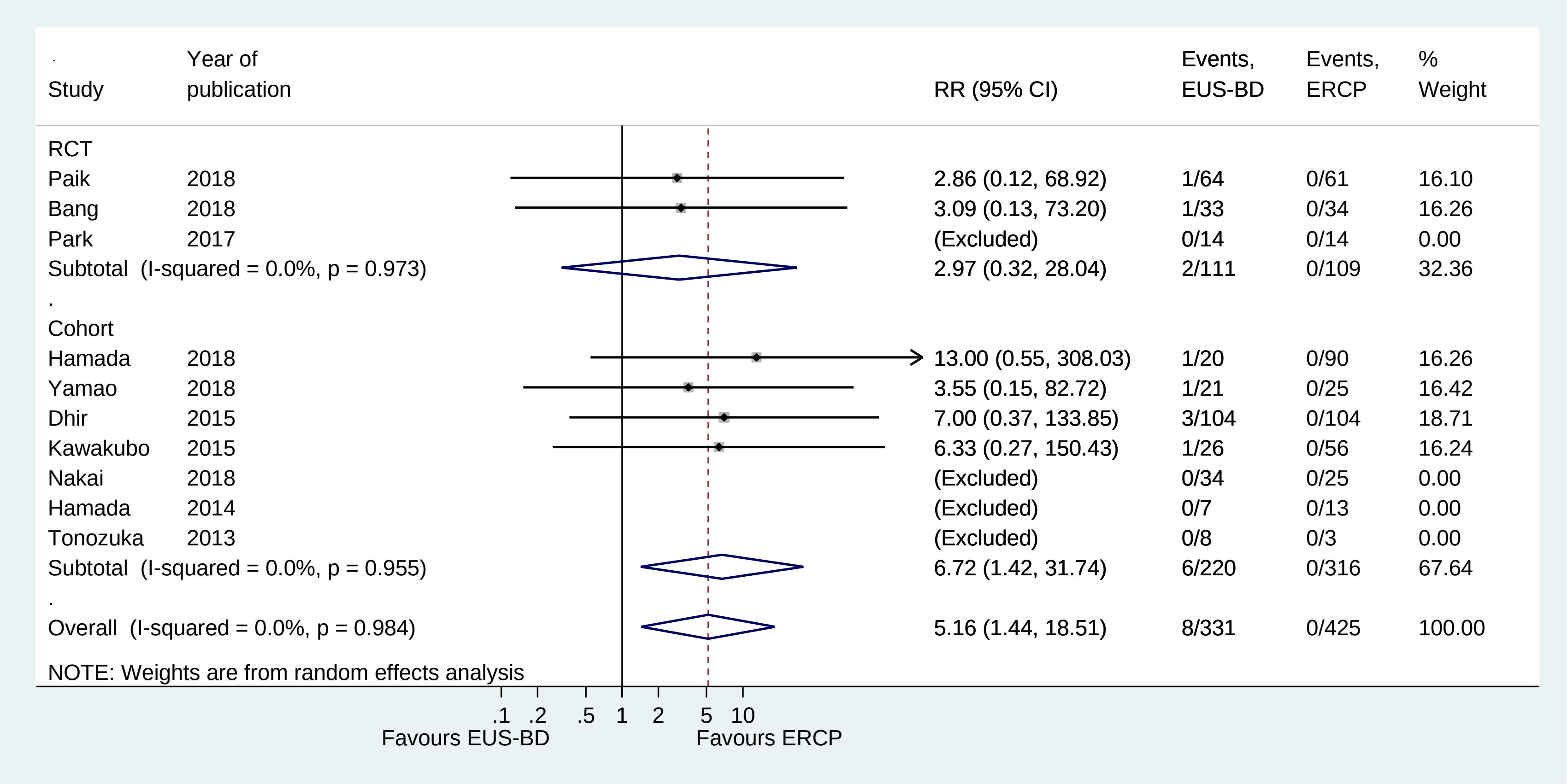

Supplementary Figure 2. Forest plot comparing bile peritonitis

RR, risk ratio; CI, confidence interval; EUS-BD, endoscopic ultrasonography-guided biliary drainage; ERCP, endoscopic retrograde cholangiopancreatography; RCT, randomized controlled trial

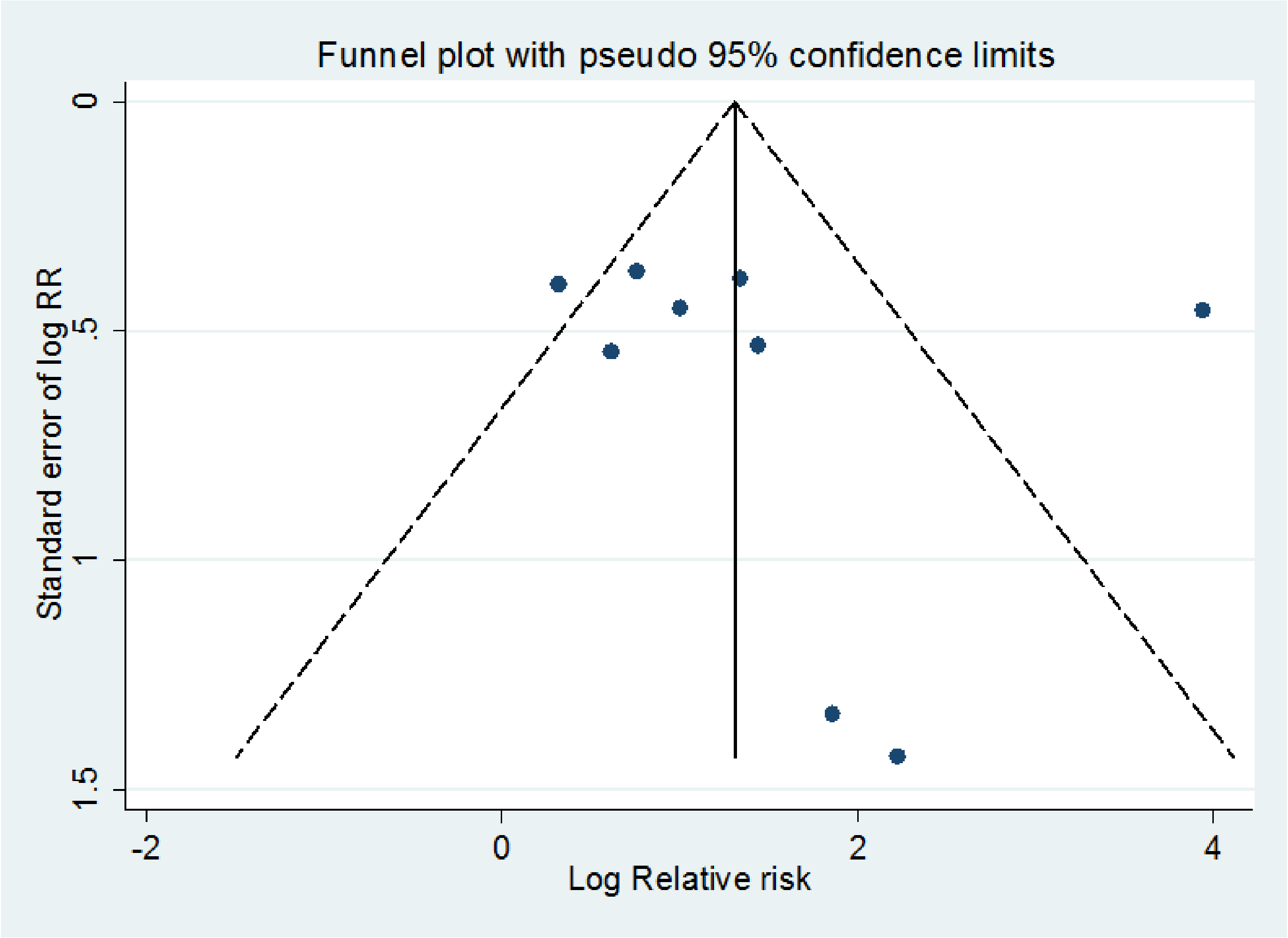

Supplementary Figure 3. Funnel plot for evaluating publication bias for adverse events

RR, risk ratio

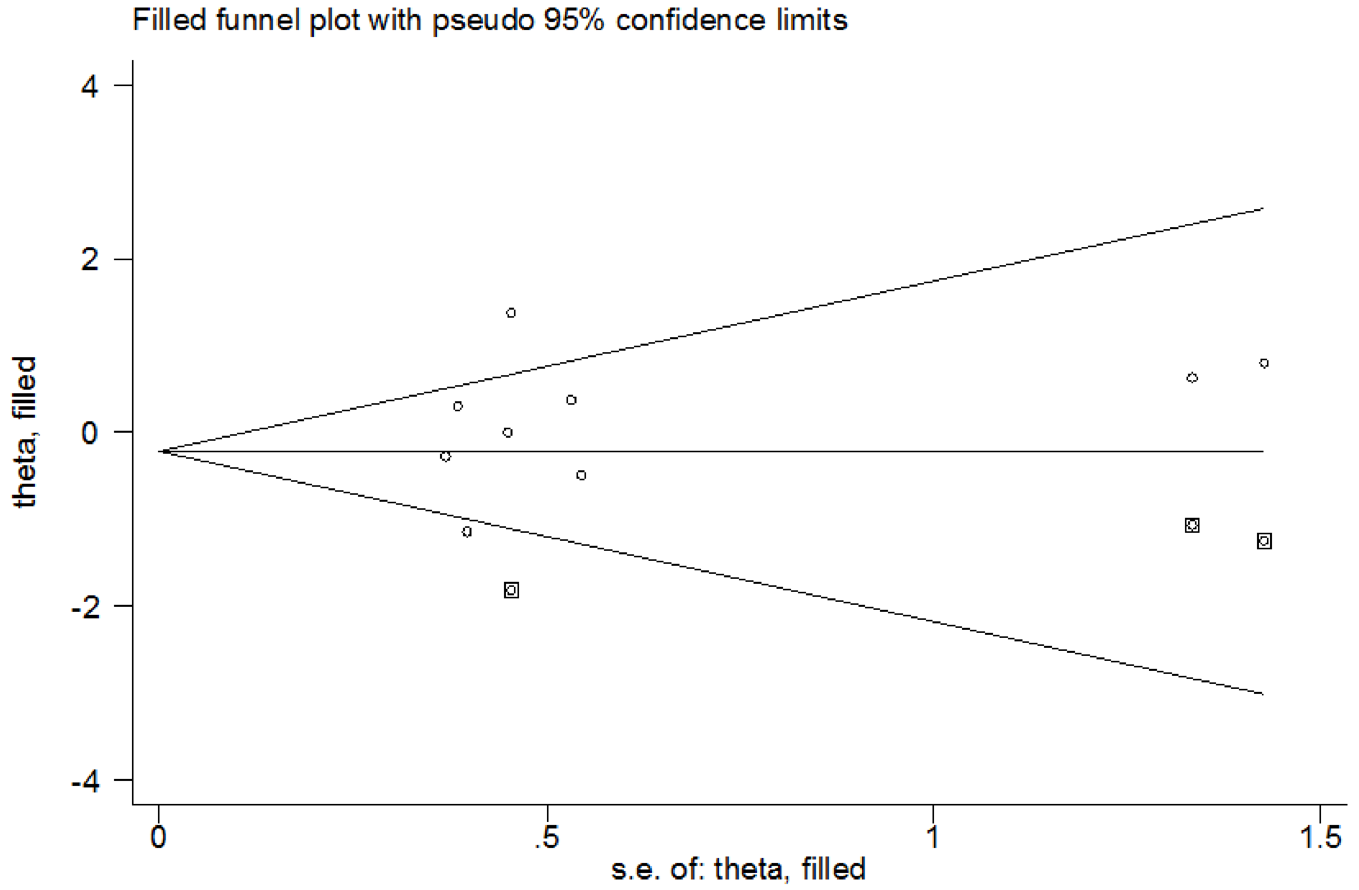

Supplementary Figure 4. Trim and fill method for evaluating publication bias for adverse events

s.e., standard error

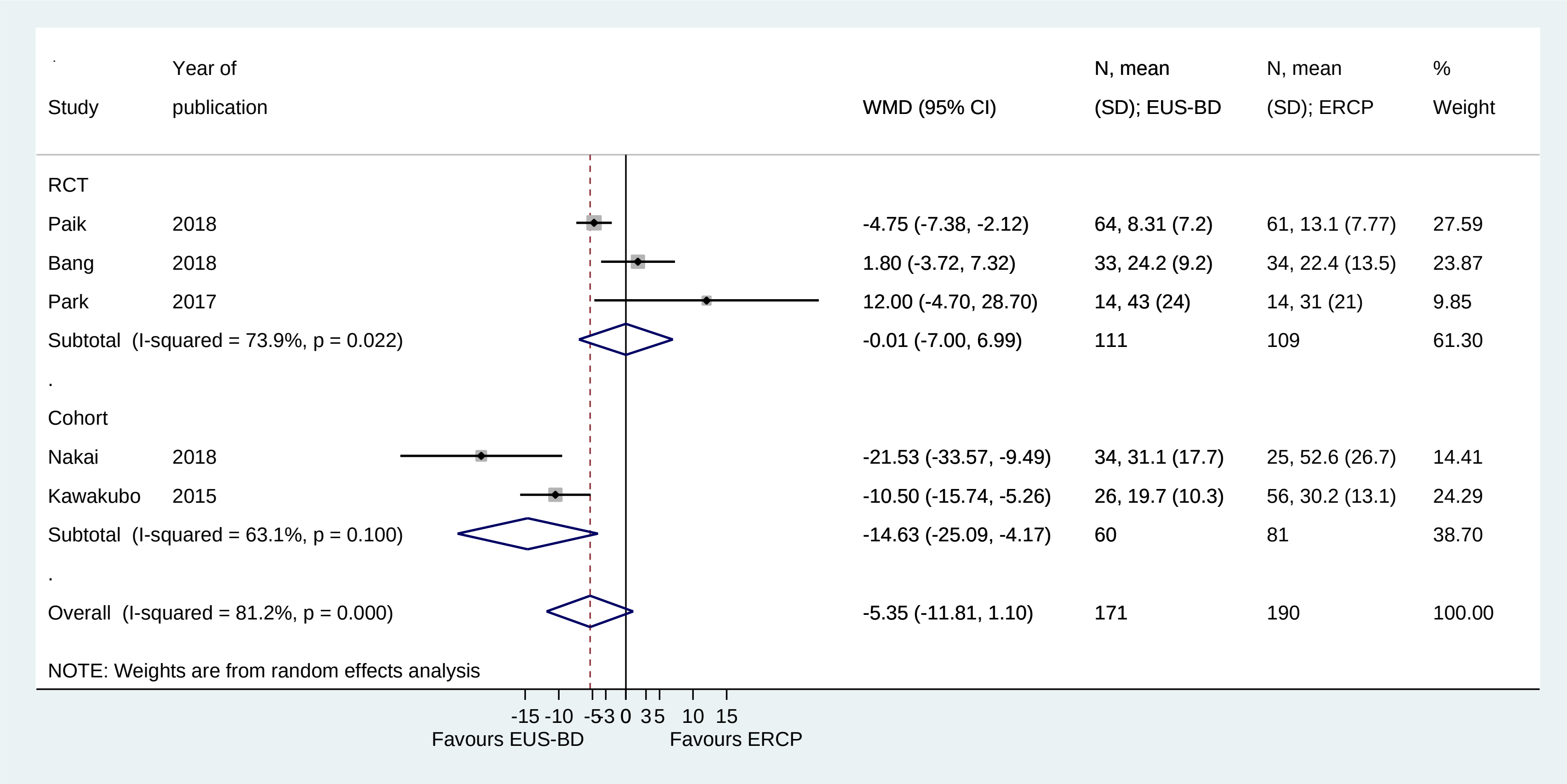

Supplementary Figure 5. Forest plot comparing procedure time

WMD, weighted mean difference; SD, standard deviation; EUS-BD, endoscopic ultrasonography-guided biliary drainage; ERCP, endoscopic retrograde cholangiopancreatography; RCT, randomized controlled trial

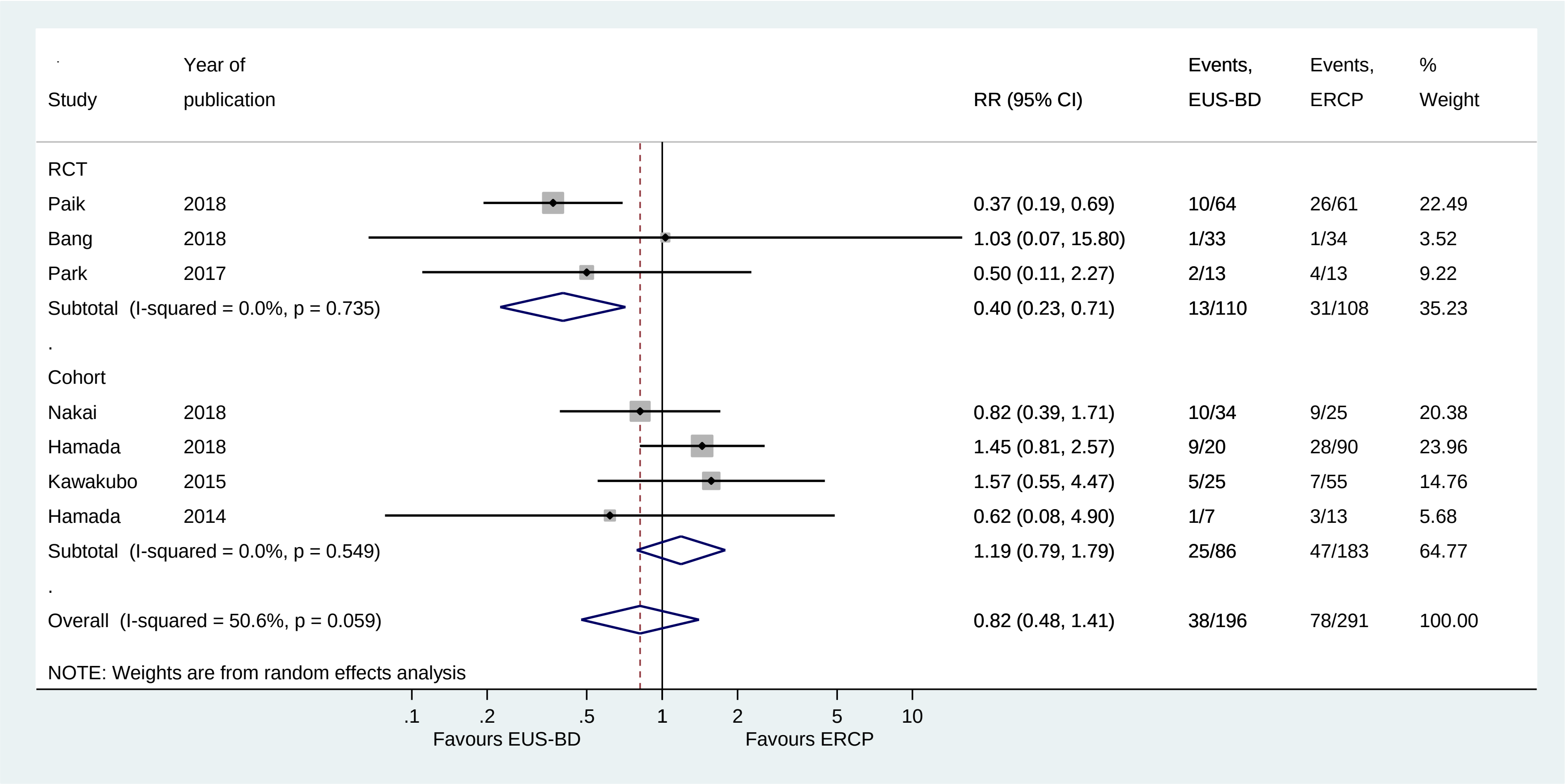

Supplementary Figure 6. Forest plot comparing reintervention rate

RR, risk ratio; CI, confidence interval; EUS-BD, endoscopic ultrasonography-guided biliary drainage; ERCP, endoscopic retrograde cholangiopancreatography; RCT, randomized controlled trial

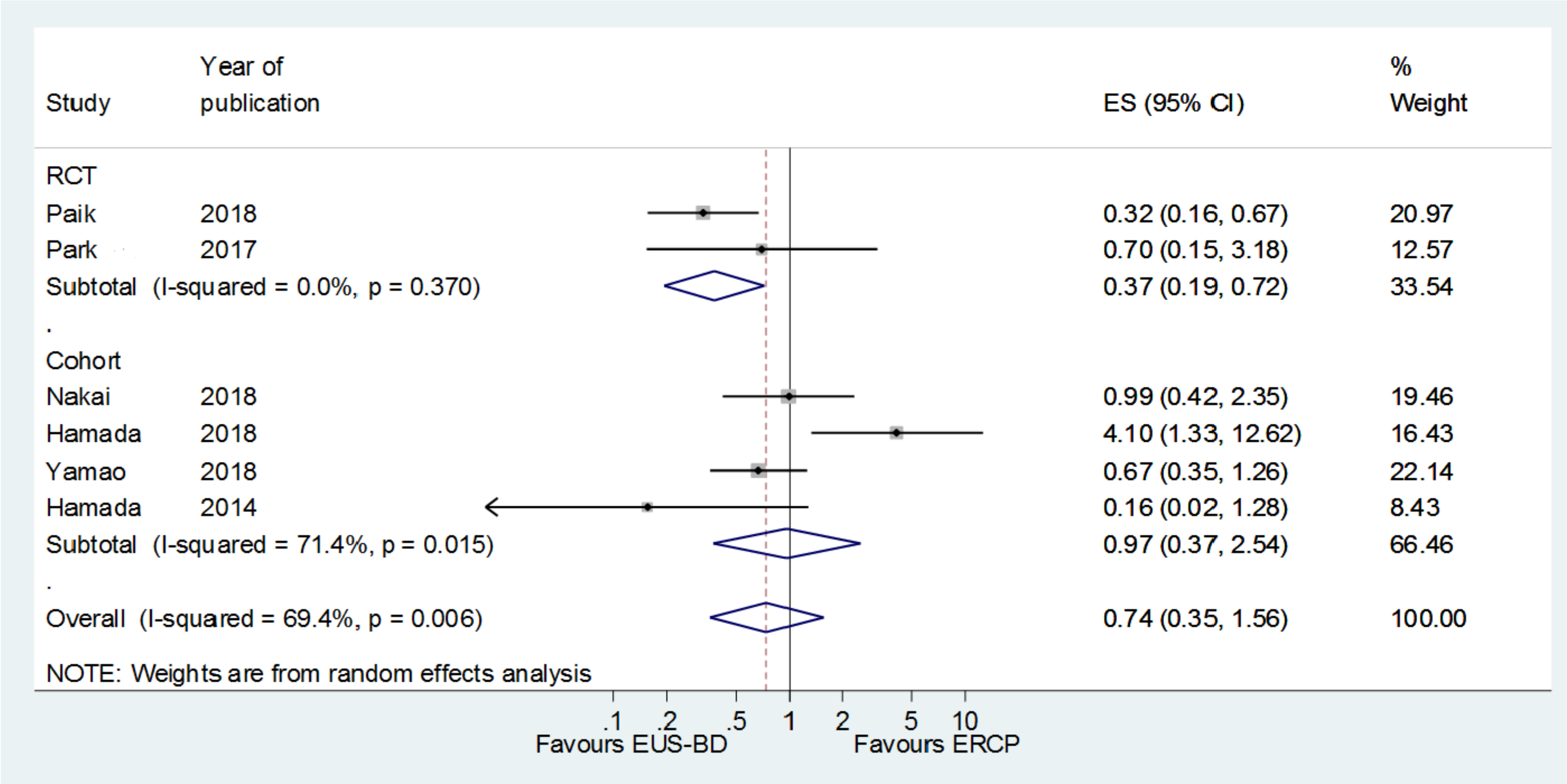

Supplementary Figure 7. Forest plot comparing stent patency

ES, effect size; CI, confidence interval; RCT, randomized controlled trial
